# The impact of corrected field output factors based on IAEA/AAPM code of practice on small‐field dosimetry to the calculated monitor unit in eclipse™ treatment planning system

**DOI:** 10.1002/acm2.12855

**Published:** 2020-04-01

**Authors:** Sammuel Mamesa, Sornjarod Oonsiri, Taweap Sanghangthum, Sumalee Yabsantia, Sivalee Suriyapee

**Affiliations:** ^1^ Medical Physics Program Department of Radiology Faculty of Medicine Chulalongkorn University Bangkok Thailand; ^2^ Division of Radiation Oncology Department of Radiology King Chulalongkorn Memorial Hospital Bangkok Thailand

**Keywords:** eclipse™ TPS, monitor unit, small field commissioning, small field detectors, small-field dosimetry, TRS 483

## Abstract

The objective of this study was to investigate the effect of field output factors (FOFs) according to the current protocol for small‐field dosimetry in conjunction to treatment planning system (TPS) commissioning. The calculated monitor unit (MU) for intensity‐modulated radiation therapy (IMRT) and volumetric modulated arc therapy (VMAT) plans in Eclipse™ TPS were observed. Micro ion chamber (0.01 CC) (CC01), photon field diode (shielded diode) (PFD), and electron field diode (unshielded diode) (EFD) were used to measure percentage depth doses, beam profiles, and FOFs from 1 × 1 cm^2^ to 10 × 10 cm^2^ field sizes of 6 MV photon beams. CC01 illustrated the highest percentage depth doses at 10 cm depth while EFD exhibited the lowest with the difference of 1.6% at 1 × 1 cm^2^. CC01 also produced slightly broader penumbra, the difference with other detectors was within 1 mm. For uncorrected FOF of three detectors, the maximum percent standard deviation (%SD) was 5.4% at 1 × 1 cm^2^ field size. When the correction factors were applied, this value dropped to 2.7%. For the calculated MU in symmetric field sizes, beam commissioning group from uncorrected FOF demonstrated maximum %SD of 6.0% at 1 × 1 cm^2^ field size. This value decreased to 2.2% when the corrected FOF was integrated. For the calculated MU in IMRT‐SRS plans, the impact of corrected FOF reduced the maximum %SD from 6.0% to 2.5% in planning target volume (PTV) less than 0.5 cm^3^. Beam commissioning using corrected FOF also decreased %SD for VMAT‐SRS plans, although it was less pronounced in comparison to other treatment planning techniques, since the %SD remained less than 2%. The use of FOFs based on IAEA/AAPM TRS 483 has been proven in this research to reduce the discrepancy of calculated MU among three beam commissioning datasets in Eclipse™ TPS. The dose measurement of both symmetric field and clinical cases comparing to the calculation illustrated the dependence of the types of detector commissioning and the algorithm of the treatment planning for small field size.

## 1 INTRODUCTION

The trend of utilizing the concept of small field in radiation therapy has been tremendously increasing over the past decades.^1^, ^2^ However, small‐field dosimetry presents several challenges due to three major problems. The first is loss of lateral charged particle equilibrium (LCPE). This problem occurs when the beam half width or beam radius is getting smaller than the range of lateral charged particle equilibrium (r*_LCPE_*). The second problem is partial source occlusion from collimating devices which yields to the larger full width at half maximum (FWHM) of dose profiles than the actual field size setting.^2^ Both problems are beam related conditions, while the third obstacle is associated to the choice of appropriate detectors. Detectors with sensitive volume smaller than the beam radius are required as they are able to measure the dosimetric characteristics in small field at high spatial resolution.^3^, ^4^ Previous work reported disagreement within 14% among various types of detectors to determine the small field output factors (FOFs).^5^ Selection of suitable detectors for small field output measurement becomes clearly cumbersome.^5^, ^6^

Several detectors such as ionization chambers and diode detectors have been developed specifically in accordance to the demand of small‐field dosimetry. The ionization chamber, which is commonly used in external beam radiotherapy provides the dose rate and energy independence. Nevertheless, ion chamber is found to underestimate the beam output, as a consequence of volume averaging effect. Volume averaging effect is an effect related to the corresponding signal from detector relative to mean absorbed dose over its sensitive volume. When the active volume of detector is larger than the beam radius, detector signal will be averaged incorrectly over its sensitive volume and eventually leads to the underestimation of measured dose.^7^, ^8^ Besides volume averaging, the perturbation of charged particle fluence due to the presence of a detector is an important issue and it must be noted that both effects are always entangled.^2^ In contrast, diode detectors have been reported as the promising detectors for small field.^9^ Diode detectors possess small active volume, excellent spatial resolution, and high sensitivity. However, the angular and energy dependence become the pitfalls when using diode.^1^, ^9^ The existence of encapsulating material with high atomic number and density also introduces another problem with respect to perturbation effect (backscattering from metallic electrode).^8^, ^9^, ^10^, ^11^

Prior to the establishment of new Code of Practice on small‐field dosimetry, Alfonso et al.^12^ proposed a new systematic approach to determine the absorbed dose in water for small and nonstandard clinical fields ($D_{w,Q_{\mathrm{clin}}}^{f_{\mathrm{clin}}}$) as shown in [eq. (1)]:

(1) $$D_{w,Q_{\mathrm{clin}}}^{f_{\mathrm{clin}}}=D_{w,Q_{\mathrm{msr}}}^{f_{\mathrm{msr}}}\Omega_{Q_{clin,}Q_{\mathrm{msr}}}^{f_{clin,}f_{\mathrm{msr}}}$$

where the $D_{w,Q_{\mathrm{msr}}}^{f_{\mathrm{msr}}}$ refers to the absorbed dose in water for the machine specific reference field size ($f_{\mathrm{msr}}$) with beam quality $Q_{\mathrm{msr}}$. The quantity of FOFs ($\Omega_{Q_{\mathrm{clin}}Q_{\mathrm{msr}}}^{f_{\mathrm{clin}}f_{\mathrm{msr}}}$) was introduced as a ratio of detector reading at the clinical field size ($M_{Q_{\mathrm{clin}}}^{f_{\mathrm{clin}}}$) to detector reading at the machine specific reference field size ($M_{Q_{\mathrm{msr}}}^{f_{\mathrm{msr}}}$). Afterward, the field output correction factors ($k_{Q_{\mathrm{clin}}Q_{\mathrm{msr}}}^{f_{\mathrm{clin}}f_{\mathrm{msr}}}$) should be implemented according to [eq. (2)]:

(2) $$\Omega_{Q_{clin,}Q_{\mathrm{msr}}}^{f_{clin,}f_{\mathrm{msr}}}=\frac{M_{Q_{\mathrm{clin}}}^{f_{\mathrm{clin}}}}{M_{Q_{\mathrm{msr}}}^{f_{\mathrm{msr}}}}k_{Q_{clin,}Q_{\mathrm{msr}}}^{f_{clin,}f_{\mathrm{msr}}}.$$

The quantity $k_{Q_{clin,}Q_{\mathrm{msr}}}^{f_{clin,}f_{\mathrm{msr}}}$ takes into account the difference between detector reading in clinical field and machine specific reference field. This factor accounts any influences from volume averaging effect, as well as perturbation effect.^11^ By using eqs. (1) and (2), the field output correction factors could be empirically written as follows:

(3) $$k_{Q_{clin,}Q_{\mathrm{msr}}}^{f_{clin,}f_{\mathrm{msr}}}=\frac{D_{w,Q_{\mathrm{clin}}}^{f_{\mathrm{clin}}}/M_{Q_{\mathrm{clin}}}^{f_{\mathrm{clin}}}}{D_{w,Q_{\mathrm{msr}}}^{f_{\mathrm{msr}}}/M_{Q_{\mathrm{msr}}}^{f_{\mathrm{msr}}}}.$$

Following aforementioned formula, effort to derive the correction factors has been accomplished by several studies. Hamza et al. reported the output correction factors for nine detectors covering the air and liquid ion chambers, silicon diodes, and diamond detectors.^8^ Azangwe et al. calculated the correction factors for a wide range of real‐time detectors and passive detectors.^11^ Correction factors cannot be ignored to obtain the accurate small FOFs. Improper selection of detectors along with neglecting use of correction factors have been reported as the main causes of pathetic accident to patients undergoing stereotactic radiation therapy.^13^

Another attention related to the small‐field external beam radiotherapy is the accuracy of output factors where the dose computation algorithm holds pivotal role on that. Two types of dose computation algorithms are available in radiotherapy TPS, namely type “a” and type “b” algorithms.^14^ The algorithm of type “a” is correction based algorithm, where it strongly depends on the attenuation process. For type “b” algorithm, the calculation algorithm is model based algorithm to estimate the scatter and secondary electrons, which is essential to obtain an accurate dose estimation in small field.^15^ The examples of type “b” algorithm are Anisotropic Analytical Algorithm (AAA) and Acuros XB (AXB).

Both algorithms are implemented in Varian Eclipse™ TPS (Varian Medical Systems, Palo Alto, CA, USA). Numerous studies have shown better dose computation of AXB than AAA, especially in heterogeneous medium.^16^, ^17^ AXB is based on the analytical solution of the Boltzman radiation transport equation and takes into account the chemical composition of each material in the volume during radiation transport in the medium. Previous studies also reported comparable outcomes between AXB and Monte Carlo simulation.^18^, ^19^

In respect to the accuracy of dose calculation algorithm for small field, Kron et al. tested AAA and AXB to compute the dose in volumetric modulated arc therapy (VMAT), where the small fields were segmented using multileaf collimator (MLC). Measurements were performed in a homogeneous medium with the use of single detector, PTW natural diamond (PTW GmbH, Freiburg, Germany).^20^ The authors discovered that both algorithms predicted the correct dose computation, as long as the beam modeling parameters were adequately tuned, particularly the effective spot size (ESS). Evaluation of dose calculation accuracy between both algorithms likewise has been conducted by Fogliata et al. against the experimental measurement using PTW microDiamond (PTW GmbH, Freiburg, Germany), with special attention given to the optimal setting for dose calculation algorithm ^15^.

Preceeding reports, however, did not compartmentalize the measured output factors from various types of detectors which were advised in many literatures for small‐field dosimetry.^4^, ^9^ Meanwhile, Garcia Garduno et al. scrutinized the impact of using six different detectors to commission 6 MV photon beams of Novalis Linear Accelerator. The evaluation of TPS was made in iPlan® TPS which employed Clarkson‐type algorithm (Physics Manual, BrainLab, Germany). Their work only covered the dose distributions in three cases of brain SRS without concerned to the calculated monitor unit (MU) in TPS.^21^

The calculated MU in TPS reflects the dose delivery to patients. Before TPS is ready for clinical use, commissioning should be performed by entering the measured FOFs into TPS.^22^ From this viewpoint, it is clear to see the bond between both quantities. Incorrectly measured output factors could bring an inaccurate calculated MU in TPS, which eventually jeopardize patient safety.^23^ To the best of authors' knowledge, attention to evaluate the calculated MU in small field has not been made so far.

Recently, the first publication dedicated for small‐field dosimetry used in external beam radiotherapy entitled Technical Reports Series (TRS) number 483 has been issued by IAEA in cooperation with AAPM Therapy Physics Committee.^2^ The current protocol introduces and classifies the field output correction factor ($k_{Q_{\mathrm{clin}}Q_{\mathrm{msr}}}^{f_{\mathrm{clin}}f_{\mathrm{msr}}}$) according to types of recommended detectors, the equivalent square field sizes (S_clin_), energy of the beam, as well as types of the equipments (Cyber Knife, Gamma Knife, Linear Accelerator, and TomoTherapy).

In response to the establishment of current protocol for small‐field dosimetry, the present study aims to address the effort for exploring the calculated MU in Eclipse™ TPS where the field output are collected from various types of detectors with the use of field output correction factors ($k_{Q_{\mathrm{clin}}Q_{\mathrm{msr}}}^{f_{\mathrm{clin}}f_{\mathrm{msr}}}$) according to the current protocol. The comparison of calculated and measured output factors were presented for the commissioning of each detector, both corrected and uncorrected FOFs. The measurement of isodose distribution in the small SRS cases compared with the calculations were also included.

## 2 MATERIALS AND METHODS

### 2.A Experimental measurements

This research was started with the measurements of percentage depth doses, beam profiles, and determination of FOFs from 6 MV flattened photon beams (TPR_20,10_ = 0.665) generated by Varian TrueBeam™ Linear Accelerator (Varian Medical Systems, Palo Alto, CA, USA). There were three main detectors used in this study: IBA micro ion chamber (0.01 CC) (CC01) microionization chamber, IBA photon field diode (shielded diode) (PFD) shielded diode, and IBA electron field diode (unshielded diode) (EFD) unshielded diode (IBA Dosimetry, Schwarzenbruck, Germany). The specifications of all detectors including IBA CC13 and EDGE detector (Sun Nuclear Corporation, Melbourne, FL) which were used for the previous commissioning data in the clinic are summarized in Tables 1 and 2. All measurements were undertaken in IBA Blue water phantom. The field sizes were segmented using jaw setting.

**TABLE 1:** Characteristics of ionization chambers.

Detector	Cavity volume (cm^3^)	Cavity length (mm)	Wall material	Wall thickness (g/cm^2^)	Central electrode material
IBA CC01	0.01	3.6	C‐552	0.088	Steel
IBA CC13	0.13	5.8	C‐552	0.070	C‐552

**TABLE 2:** Characteristics of diode detectors.

Detector	Sensitive volume (mm^3^)	Diameter of side length of sensitive area (mm)	Geometric form of sensitive area	Thickness of sensitive area(mm)	Shielded
IBA PFD	0.190	2.0	Disc	0.06	Yes
IBA EFD	0.190	2.0	Disc	0.06	No
EDGE	0.019	0.8	Square	0.03	Yes

### 2.B Percentage depth doses and beam profiles

Scanning of percentage depth doses and beam profiles were carried out on symmetric field sizes from 1 × 1 cm^2^, 2 × 2 cm^2^, 3 × 3 cm^2^, 4 × 4 cm^2^, 6 × 6 cm^2^, and 10 × 10 cm^2^ by CC01, PFD, and EFD detectors. Beam scanning was performed from 310 mm depth to the surface of water phantom. Meanwhile, beam profiles were scanned at depth of maximum dose (d*_max_*), 5, 10, 20, and 30 cm depth. Source to surface distance (SSD) was 100 cm. CC01 was set in perpendicular direction to the central beam axis according to the guidelines.^24^ PFD and EFD were set in parallel direction to beam axis and the effective point of measurement was at its surface of detector's active volume. No reference detector was utilized during beam scanning. The scanning speed was 0.3 cm/sec to minimize any effect from water ripple which disrupt our measurements. Smoothing of percentage depth doses and beam profiles in all field sizes were accomplished using IBA OmniPro Accept software. Since the intention of this work was to use three different detectors for small field commissioning, the assessment was made to compare the measured percentage depth doses and beam profiles using each detector to our previous commissioning dataset. For previous commissioning dataset that is used in routine clinical practice (reference data), IBA CC13 ionization chamber was employed for percentage depth doses, beam profiles, and relative output measurements from 4 × 4 cm^2^ to 40 × 40 cm^2^ field sizes, while from 2 × 2 cm^2^ to 3 × 3 cm^2^ field sizes, beam scanning and relative output measurements were performed using EDGE detector with its sensitive volume of 0.019 mm^3^. Varian recommended the smallest field size of 3 × 3 cm^2^ for commissioning; however, the smaller field size down to 2 × 2 cm^2^ could be inserted. For field size less than 2 × 2 cm^2^, the eclipse extrapolation of all parameters was applied for the dose calculation.

### 2.C Equivalent square field size

The IAEA/AAPM TRS 483 recommended that the radiation field size is defined by FWHM of the lateral beam profile measured at 10 cm depth. The measurement of radiation field was performed using EDGE detector with 100 cm SSD at 10 cm depth. The beam profiles were scanned in IBA blue water phantom from 1 × 1 cm^2^, 2 × 2 cm^2^, 3 × 3 cm^2^, 4 × 4 cm^2^, 6 × 6 cm^2^, and 10 × 10 cm^2^ field sizes in both cross and in‐plane directions. The dosimetric field width at 50% of relative dose (FWHM) from the cross (A) and in**‐**plane (B) direction was recorded. Then, the equivalent square field size (S_clin_) was calculated following equation 4 as follows:

(4) $$S_{\mathrm{clin}}=\sqrt{\mathrm{AB}}$$

S_clin_ was recommended to determine the field output correction factors according to TRS 483

### 2.D Field output factors ($\Omega_{Q_{\mathrm{clin}}Q_{\mathrm{msr}}}^{f_{\mathrm{clin}}f_{\mathrm{msr}}}$)

Determination of FOF was divided into two groups: uncorrected FOF (ratio of detector reading at any field sizes to reference field size) and corrected FOF. Measurements were conducted in various collimator field sizes: 1 × 1 cm^2^, 2 × 2 cm^2^, 3 × 3 cm^2^, 4 × 4 cm^2^, 6 × 6 cm^2^, and 10 × 10 cm^2^. The SSD and reference depth were 100 cm and 10 cm, respectively. The output reading from each field size was normalized to the output acquired at 10 × 10 cm^2^ machine specific reference field size ($f_{\mathrm{msr}}$). IBA DOSE‐1 electrometer was connected to each detector (CC01, PFD, and EFD) to measure the collected charge. For the first group, the use of field output correction factors ($k_{Q_{\mathrm{clin}}Q_{\mathrm{msr}}}^{f_{\mathrm{clin}}f_{\mathrm{msr}}}$) based on IAEA/AAPM TRS 483 were omitted. For the second group, the field output correction factors from IAEA/AAPM TRS 483 were applied using equivalent square field size $\left(S_{\mathrm{clin}}\right)\;\text{and calculated}$ and calculated following [eq. (2)].

### 2.E Dose computation in Eclipse™ treatment planning system and verification

The second step in this research was computing the corrected FOF and uncorrected FOF for each commissioning detector type of the symmetric field in the TPS. Acuros XB algorithm was chosen to compute the measured data. For percentage depth doses, beam profiles, and output factors: the measured data from each detector mentioned above were entered into the TPS from 2 × 2 cm^2^ until the field size of 6 × 6 cm^2^. The heterogeneity correction was turned on and the dose reporting mode was set into the dose to medium (D_m_). The smallest calculation grid size of 0.125 cm was applied during configuration to ensure the highest accuracy of dose calculation in small field. The effective spot size (ESS) was set to 1 mm for both X and Y directions in order to improve the dose calculation, as per the recommendation from Kron et al.^20^ Beam configuration in our study generated two main groups of beam datasets. The first group was beam configuration from uncorrected FOF, which were classified into uncorrected CC01, uncorrected PFD, and uncorrected EFD. The second group was beam commissioning datasets from the corrected FOF, which were categorized into corrected CC01, corrected PFD, and corrected EFD. Then, the outcomes of the symmetric field TPS calculation were compared with measurement for each detector type.

### 2.F Comparison of calculated MU among commissioning datasets

Observation of calculated MU in Eclipse™ was undertaken in 5 symmetric field sizes and 10 brain stereotactic radiosurgery (SRS) cases treated using intensity‐modulated radiation therapy (IMRT) and VMAT techniques. All brain SRS cases in our study were selected from clinical databases. Those plans employed 9 fields of IMRT technique and 2 arcs of gantry rotation in VMAT technique. Volumes and prescribed doses from all cases are listed in Table 3. The volume of brain tumors ranged from 0.36 to 14.06 cm^3^ with the prescribed doses ranged from 12.5 to 24 Gy and modulation factor ranged from 2.88 to 4.60 for corrected FOF in IMRT plans. The modulation factors of each case for uncorrected and corrected FOF were comparable. For symmetric field sizes, comparison of MU was explored using virtual water phantom in Eclipse™ from 1 × 1 cm^2^ to 6 × 6 cm^2^ field sizes. Prescribed dose of 1 Gy was delivered to a point of 10 cm depth in the virtual water phantom. The virtual water phantom was defined assigning the homogeneous medium with 0 Hounsfield Unit (HU). None of the parameters were changed except the beam commissioning dataset. In a similar fashion to MU comparison in symmetric field sizes, the comparison of MU in IMRT and VMAT techniques also kept all parameters unchanged including the prescribed dose, plan optimization, and dose constraints, as well as the gantry and collimator rotations. The only altered parameter was the input of beam commissioning dataset. Once the plan was executed, the calculated MU was recorded according to each commissioning data. Afterward, the %SD of calculated MU from each commissioning group (uncorrected FOF and corrected FOF) was determined following [eq.  (5)].

(5) $$\%\;\text{Standard}\;\text{deviation}\left(\text{SD}\right)=\frac{\text{SD}}{\text{Mean}}\times 100\%$$

**TABLE 3:** Summary of 10 SRS cases of brain tumors for MU comparison in IMRT and VMAT plans.

Case Number	PTV (cm^3^)	Prescribed Dose (Gy)
1	14.06	20
2	13.03	20
3	11.16	20
4	6.25	15
5	3.01	12.5
6	2.67	18
7	1.86	18
8	1.62	24
9	0.78	12.5
10	0.36	12.5

PTV, planning target volume.

## 3 RESULTS

### 3.A Experimental measurement

#### 3.A.1 Percentage depth doses and beam profiles

**FIG. 1:**
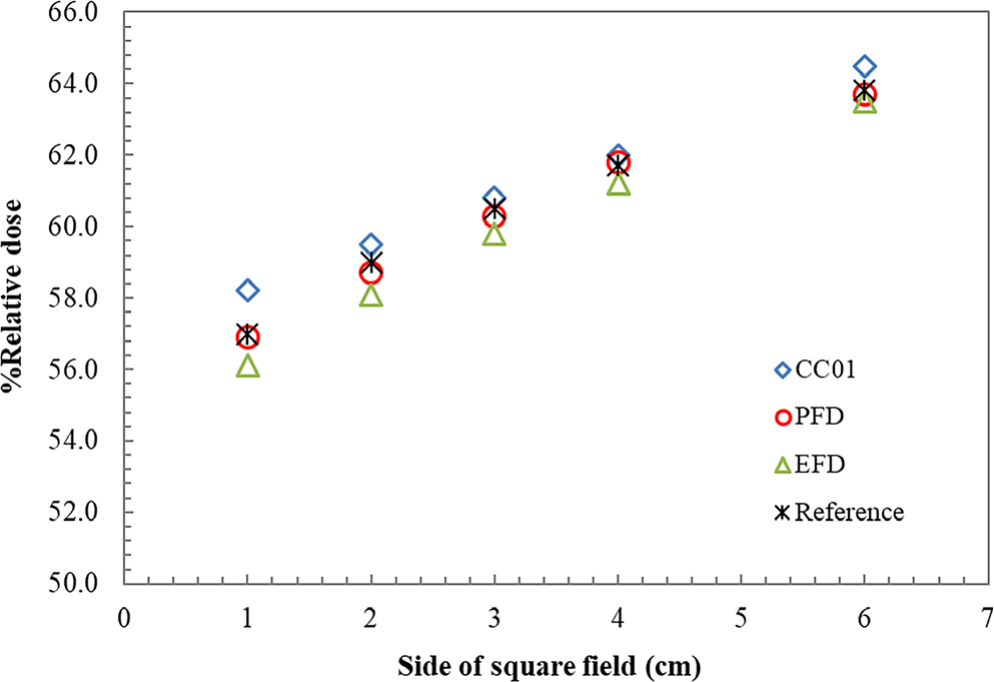
Percentage depth doses at 10 cm depth measured using CC01, PFD, and EFD in various geometric field sizes from 1 × 1 cm^2^ to 10 × 10 cm^2^.

The comparison of measured percentage depth doses at 10 cm depth for three detectors is demonstrated in Fig. 1. All detectors showed agreeable percentage depth doses at 10 × 10 cm^2^ and started to differ at 6 × 6 cm^2^, increasing more from 2 × 2 to 1 × 1 cm^2^ field sizes. CC01 demonstrated slightly higher percentage depth dose, and EFD exhibited the lowest outcome comparable to PFD. The difference between PFD and EFD slightly increased more than 1% at 2 × 2 cm^2^ and 1 × 1 cm^2^ field size. EDGE was comparable to PFD with the maximum difference of 0.3%. CC13 was comparable within 0.5% to all detectors for field size down to 3 × 3 cm^2^, except CC01 at 6 × 6 cm^2^ and EFD at 3 × 3 cm^2^ that showed difference of about 1%.

**FIG. 2:**
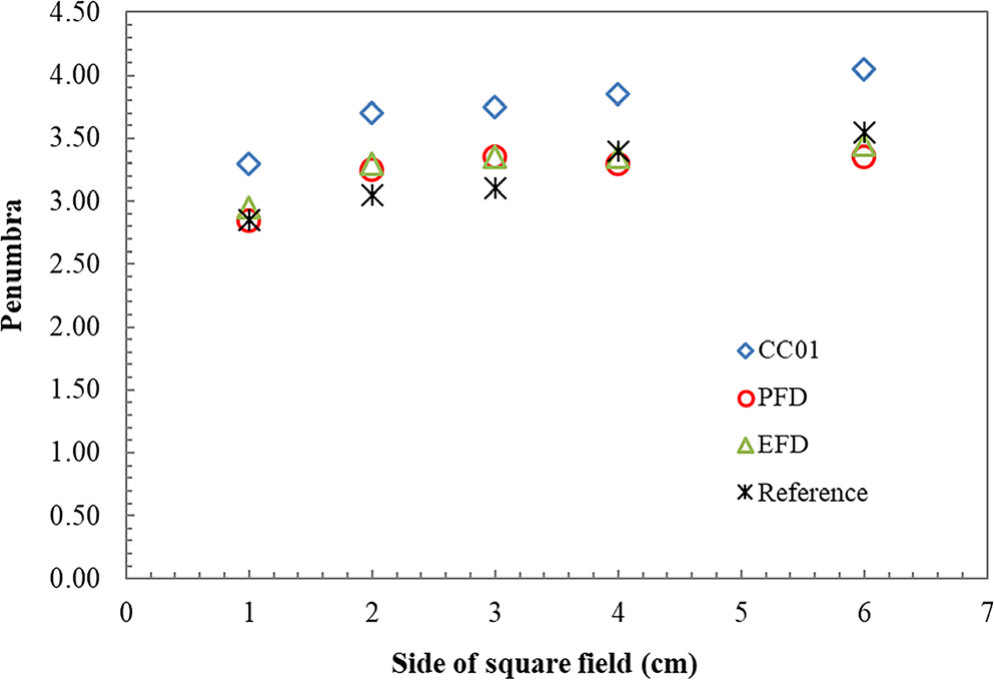
Penumbra width between 20% and 80% isodose line at depth of maximum dose (d*_max_*) measured using CC01, PFD, and EFD in various geometric field sizes from 1 × 1 cm^2^ to 10 × 10 cm^2^.

For beam profiles, the lateral distance between 20% and 80% isodose line (penumbra width) at d*_max_* was analyzed as displayed in Fig. 2. Measurement using CC01 yielded broader penumbra within 1 mm compared to measurement using diode detectors, and more pronounced as the field size decreased. In contrast, penumbra width obtained from PFD and EFD agreed well. This was due to the equal size of both diode detectors.

### 3.B Equivalent square field size

The equivalent square field S_clin_ in 10 cm under the surface of water phantom is shown in Table 4, it is used to determine the FOF correction factor according to Table 26 in TRS 483.

**TABLE 4:** The geometric field size along with the corresponding dosimetric field width in the cross‐plane and in‐plane direction measured at 10 cm depth of FWHM.

Side of collimator square field (cm) at 100 cm	Side of collimator square field (cm) at 110 cm	Dosimetric field width (Cross‐plane: cm)	Dosimetric field width (In‐plane: cm)	Side of equivalent square field, S_clin_ (cm)
6	6.6	6.45	6.63	6.54
4	4.4	4.23	4.43	4.33
3	3.3	3.12	3.32	3.22
2	2.2	2.01	2.20	2.10
1	1.1	0.90	1.11	1.00

FWHM, full width at half maximum.

### 3.C Field output factors ($\Omega_{Q_{\mathrm{clin}}Q_{\mathrm{msr}}}^{f_{\mathrm{clin}}f_{\mathrm{msr}}}$)

The FOFs for symmetric fields in this study were observed between the uncorrected and the corrected groups. Table 5 shows the uncorrected FOF of PFD, EFD, CC01, CC13, and EDGE. The uncorrected FOF from PFD were the highest among other detectors, followed by CC13 and EDGE detector which the values were comparable until 2 × 2 cm^2^ field size. EFD exhibited the lowest field output factors. The results from CC01 were between EDGE and EFD diode detectors. The %SD was considered for only three detectors of CC01, PFD, and EFD. An increase of %SD was discovered by decreasing the field size to 1 × 1 cm^2^, where the maximum %SD was 5.4%. The second measurement with the use of field output correction factors according to S_clin_ from IAEA/AAPM TRS 483^2^ is displayed in Table 6. The deviation among detectors significantly decreased after implementing the correction factors to each detector. In the smallest field size, the maximum %SD drastically reduced to 2.7%.

**TABLE 5:** Determination of uncorrected field output factors using CC01, PFD, EFD, EDGE, and CC13.

Side of collimator square field @t 100 cm SSD (cm)	CC01	PFD	EFD	Average ± SD*	%SD*	EDGE	CC13
6	0.916	0.928	0.908	0.917 ± 0.01	1.1	0.918	0.922
4	0.859	0.875	0.845	0.860 ± 0.02	1.7	0.863	0.865
3	0.824	0.844	0.811	0.826 ± 0.02	2.0	0.830	0.833
2	0.783	0.811	0.771	0.788 ± 0.02	2.6	0.794	0.790
1	0.674	0.736	0.668	0.693 ± 0.04	5.4	0.720	0.615

* Average and %SD do not consider the results from EDGE and CC13.

**TABLE 6:** Determination of field output factors using CC01, PFD, EFD, EDGE, and CC13 after correction based on IAEA/AAPM TRS 483. The corrected field output factors using CC13 and EDGE detectors are attached.

Side of collimator square field size @t 100 cm SSD (cm)	CC01	PFD	EFD	Average ± SD	%SD	EDGE	CC13
6	0.920	0.928	0.916	0.921 ± 0.01	0.7	0.918	0.922
4	0.865	0.873	0.857	0.865 ± 0.01	0.9	0.865	0.865
3	0.831	0.840	0.824	0.832 ± 0.01	1.0	0.829	0.834
2	0.790	0.800	0.782	0.791 ± 0.01	1.1	0.789	0.797

### 3.D Verification of dose computation in Eclipse™ treatment planning system and measurement

**FIG. 3:**
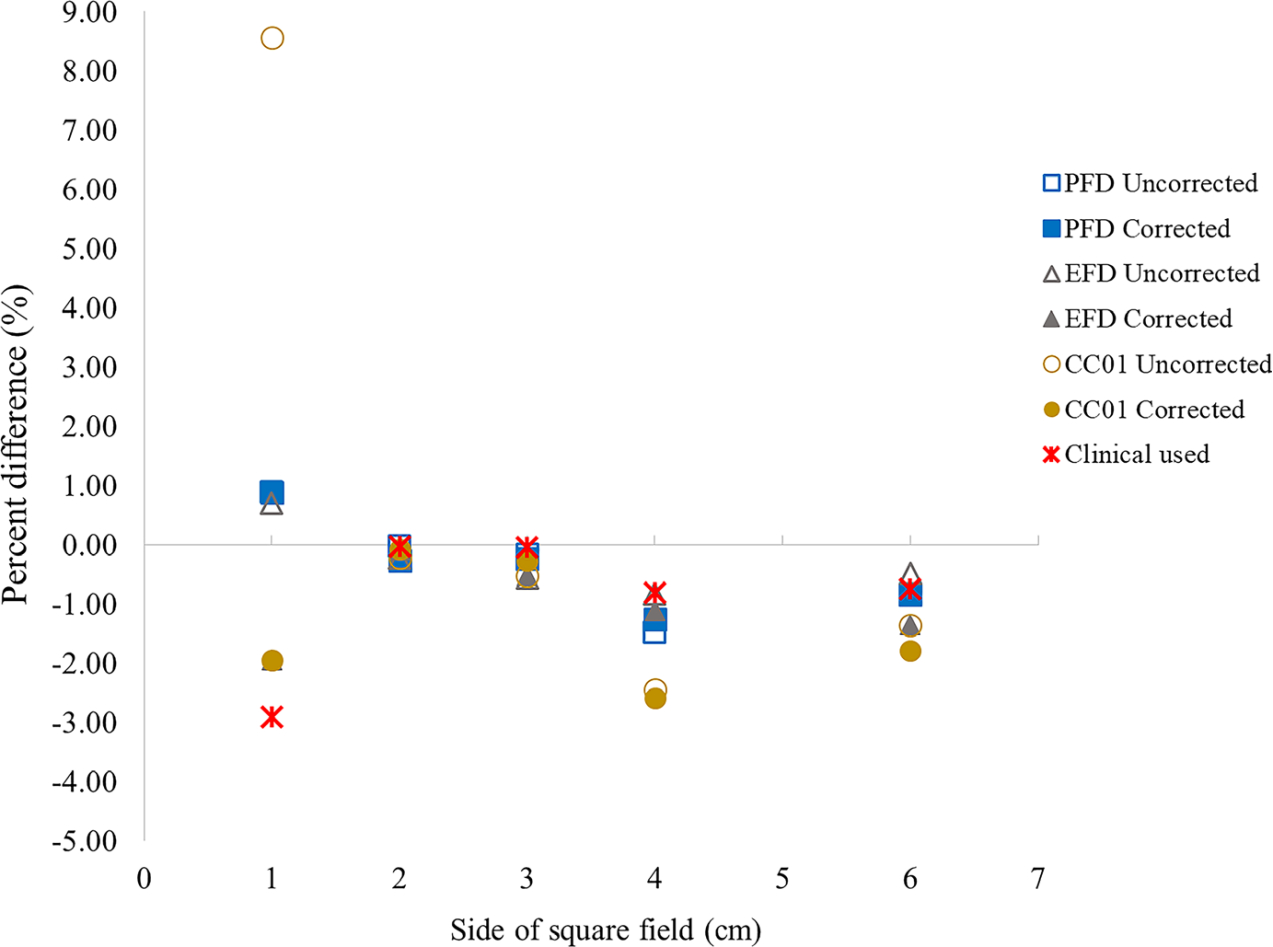
Percent difference of the field output factors for TPS calculation and the measurement for uncorrected and corrected field output factors.

The difference between TPS calculation and the measurement of symmetric fields for corrected and uncorrected FOF in three detectors and uncorrected FOF of CC13 and EDGE detectors used in clinical situation are plotted in Fig. 3. Both corrected and uncorrected FOF for three detectors and uncorrected FOF for EDGE and CC13 in clinical agreed well with the TPS calculation within 1%, for 2 × 2 cm^2^ and 3 × 3 cm^2^ field sizes, but deviated more for the larger fields of 4 × 4 cm^2^ and 6 × 6 cm^2^. When comparing the difference with the detectors in clinical use, the three detectors exhibited close agreement for 2 × 2 cm^2^ and 3 × 3 cm^2^ field sizes than the larger fields of 4 × 4 cm^2^, and 6 × 6 cm^2^. For 1 × 1 cm^2^ field size which was calculated by extrapolation data from the field of 2 × 2 cm^2^ and more, the difference between calculation and measurement was larger in the uncorrected CC01 and smaller in PFD for both corrected and uncorrected FOF.

**FIG. 4:**
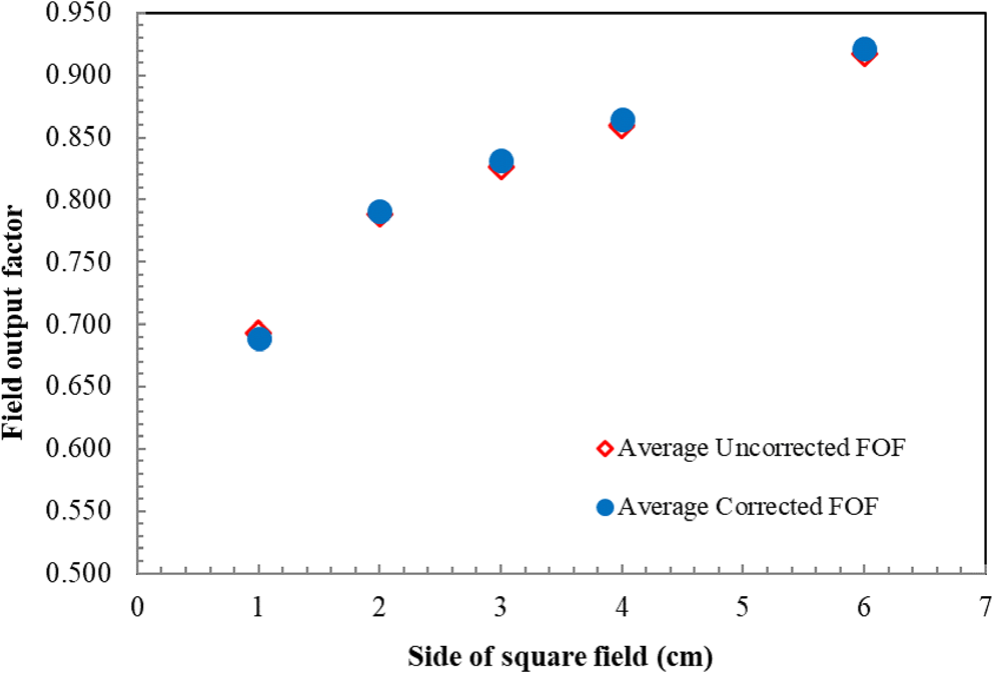
Field output factors between average uncorrected and average corrected from three detectors.

Other analysis was made by comparing the average uncorrected FOF to the average corrected FOF as shown in Fig. 4, where a strong agreement between both groups within 0.5% mean difference was discovered. It should be noted that only three detectors were involved (CC01, PFD, and EFD).

### 3.E Comparison of calculated MU among commissioning datasets

**FIG. 5:**
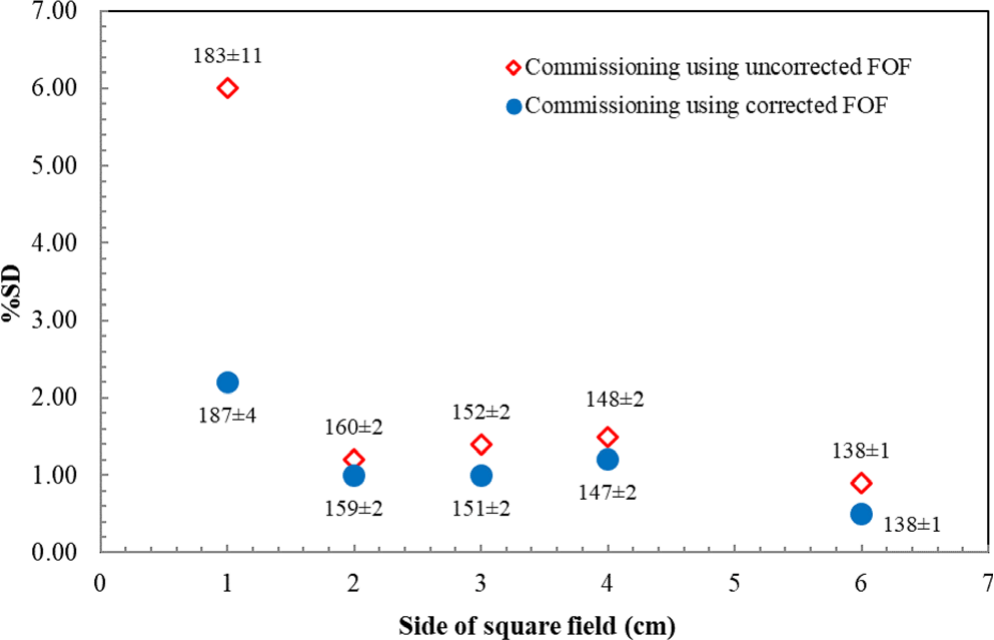
Percent SD of calculated MU from 1 × 1 cm^2^ to 6 × 6 cm^2^ field sizes between commissioning using uncorrected FOF and corrected FOF. The number of MU (average ± SD) is labelled in each commissioning dataset for each field size.

For the calculated MU in symmetric field sizes, the average values for three detectors from 2 × 2 cm^2^ to 6 × 6 cm^2^ field sizes in both uncorrected and corrected FOF were very close, except at 1 × 1 cm^2^ field size. It was apparent that commissioning from uncorrected FOF yielded %SD less than 2.0% from 2 × 2 cm^2^ to 6 × 6 cm^2^ field sizes as displayed in Fig. 5. The %SD eventually reached the largest of 6.0% (183 ± 11.0 MU) at 1 × 1 cm^2^ field size. Nevertheless, this value declined sharply to 2.2% (187 ± 4.1 MU) when commissioning using corrected FOF was applied.

**FIG. 6:**
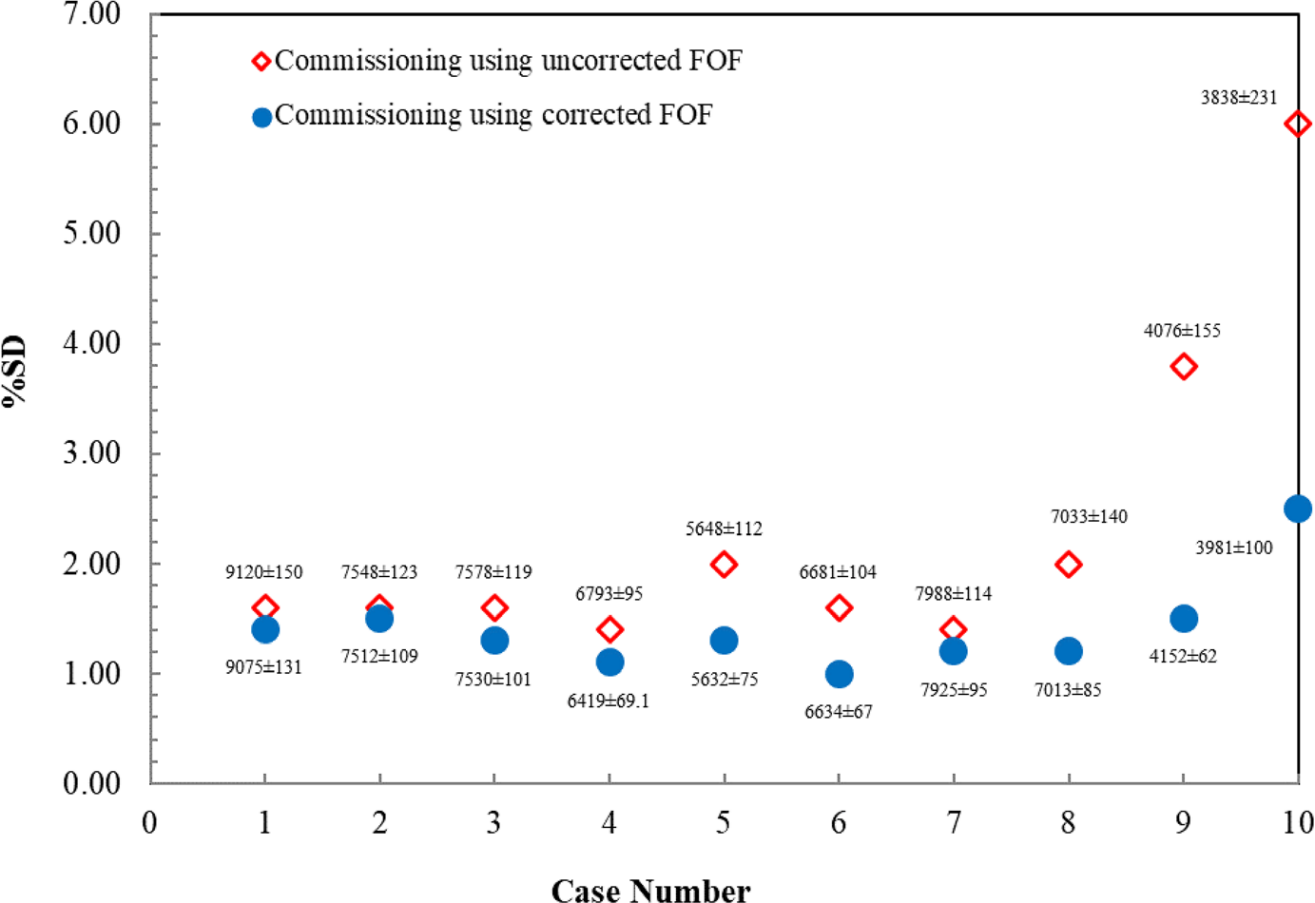
Percent SD of calculated MU between commissioning using uncorrected FOF and corrected FOF in 10 brain SRS cases treated with IMRT technique. The number of MU (average ± SD) is labelled in each commissioning dataset for each case.

**FIG. 7:**
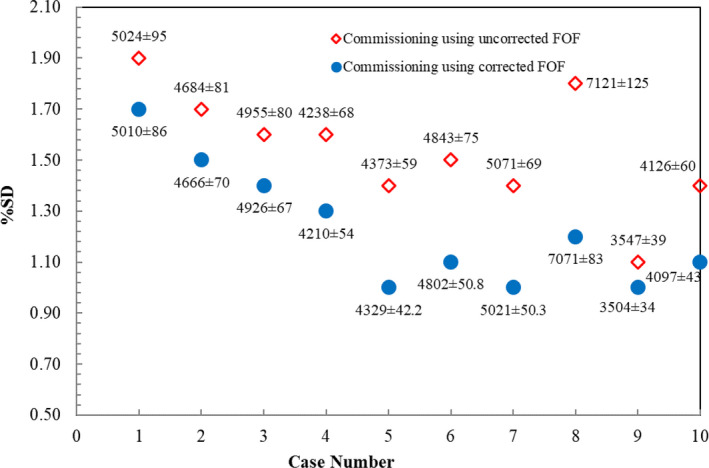
Percent SD of calculated MU between commissioning using uncorrected FOF and corrected FOF in 10 brain SRS cases treated with VMAT technique. The number of MU (average ± SD) is labelled in each commissioning dataset for each case.

For 10 IMRT‐SRS plans, commissioning from uncorrected FOF presented the %SD in the range from 1.4% to 6.0%. The %SD started to increase in case number 9 and reached the maximum percent deviation of 6.0% (3838 ± 231 MU) in case number 10 as shown in Fig. 6. When commissioning using corrected FOF was employed, the maximum %SD in case number 10 decreased to 2.5% (3981 ± 100 MU). The last observation was conducted in 10 VMAT‐SRS plans. Following the plot in Fig. 7, the %SD from both commissioning groups were less than 2.0%. The %SD from commissioning using uncorrected FOF ranged from 1.1% to 1.9%, and slightly declined to the range from 1.0% to 1.7% for commissioning using corrected FOF.

## 4 DISCUSSION

The focus of this study was to observe the effect of integrating corrected FOF based on IAEA/AAPM TRS 483 into Eclipse™ TPS with attention to the calculated MU and the measured MU for most different clinical cases. The accuracy of field output factors is one of the parameters influencing the number of MU^15^, ^25^ and dose calculation. Determination of small field output factors turns to be an important matter since the lateral charged particle disequilibrium is obvious. Selection of the appropriate detectors for small field output measurement is essential.^9^, ^11^, ^26^ In this research, three main detectors were utilized to determine the field output factors: CC01, PFD, and EFD. These detectors are recommended by IAEA/AAPM TRS 483. CC01 represents the recommended ionization chamber, while the PFD and EFD designate the recommended diode detectors. CC01 illustrated the highest percentage depth doses at 10 cm depth while EFD exhibited the lowest. The maximum difference of 1.6% was observed at 1 × 1 cm^2^ as previously shown in Fig. 1. For measured penumbra, CC01 produced slightly broader penumbra in regards to the larger size of detector. Nevertheless, the difference was only within 1 mm.

For the uncorrected FOF (ratio of reading), higher results from measurement using PFD, EDGE, and CC13 detectors were observed than the measurement using CC01 and EFD detectors. The uncorrected FOF of CC13 was lowest at 1 × 1 cm^2^ field size due to the limited of large volume. Meanwhile, the unwanted scatter from high density of encapsulating material of PFD and EDGE was the main reason of higher outcome. However, smaller size of EDGE detector makes less effect of scatter than PFD.^8^, ^9^, ^10^, ^11^ Unlike PFD, EFD detector contains no high density material of encapsulating component. This enables the EFD to minimize an over‐response from backscattering effect of shielding material.^8^, ^9^ When the FOF correction factors were applied, the three detectors come closer with the reduction of %SD. Once the measured output factors were configured to the TPS, the AXB algorithm did calculation. The tuning parameter to optimize the calculation of output factors was made by adjusting the effective spot size (ESS). ESS is the parameter associated to the geometric penumbra and partial source occlusion related to the small field condition.^27^ Following the recommendation, ESS in this study was tuned to 1 mm in X and Y direction.^15^ The tuning was performed with the same parameters for all the models.

Consider the detectors that were used for previous commissioning in the routine clinic practice without FOF correction: EDGE detector for 2 × 2 cm^2^ and 3 × 3 cm^2^ field sizes and CC13 for 4 × 4 cm^2^ up to 40 × 40 cm^2^ field sizes. The FOF correction factors for EDGE detector were close to one (0.999, 0.994 for 2 × 2 cm^2^ and 3 × 3 cm^2^ field sizes, respectively). Therefore, the corrected FOF of EDGE detector did not change much as seen in Table 6 compared to Table 5. The small size of EDGE detector would not cause more effect of scattering in the shielded material as PFD.

However, the response of diode detector in small and large field would be different, the intermediate field method (IFM) was recommended in IAEA TRS‐483 to link the response of small detector from small to large fields using CC13 for intermediate field. The intermediate FOF is shown in [eq. (6)]:

(6) $$\Omega_{Q_{clin,}Q_{\mathrm{msr}}}^{f_{clin,}f_{\mathrm{msr}}}={\left[\frac{M_{Q_{\mathrm{clin}}}^{f_{\mathrm{clin}}}}{M_{Q_{\text{int}}}^{f_{\text{int}}}}k_{Q_{clin,}Q_{\text{int}}}^{f_{clin,}f_{\text{int}}}\right]}_{\text{det}}{\left[\frac{M_{Q_{\text{int}}}^{f_{\text{int}}}}{M_{Q_{\mathrm{msr}}}^{f_{\mathrm{msr}}}}k_{Q_{\text{int},}Q_{\mathrm{msr}}}^{f_{\text{int},}f_{\mathrm{msr}}}\right]}_{\mathrm{IC}}.$$

The equation above clearly demonstrates that two output correction factors are required, one for each detector.

The data for all detectors by IFM are shown in Table 7. The agreeable outcomes for all detectors were observed. The %SD was reduced compared to the uncorrected and corrected FOF, which was only 1.7% for 1 × 1 cm^2^ field size. When the intermediate FOF of EDGE detector was compared with the uncorrected FOF, the comparable FOF for all range of field sizes were observed except 1 × 1 cm^2^ field size.

**TABLE 7:** Determination of field output factors using CC01, PFD, EFD, EDGE, and CC13 for intermediate field based on IAEA/AAPM TRS 483.

Side of collimator square field size at 100 cm SSD (cm)	CC01	PFD	EFD	Average ± SD	%SD	EDGE	CC13
6	0.920	0.919	0.925	0.922 ± 0.00	0.3	0.921 (0.918)	0.922 (0.922)
4	0.865	0.865	0.865	0.865 ± 0.00	0.0	0.865 (0.863)	0.865 (0.865)
3	0.831	0.833	0.832	0.832 ± 0.00	0.1	0.832 (0.830)	0.834 (0.833)
2	0.790	0.792	0.790	0.791 ± 0.00	0.2	0.792 (0.794)	0.797 (0.790)
1	0.686	0.701	0.678	0.689 ± 0.01	1.7	0.698 (0.720)	– (0.615)

() is the uncorrected FOF: the data of commissioning in clinical used.

The comparison of measurement and TPS calculation of symmetric field for all detectors (Fig. 3) yielded a good agreement for 2 × 2 cm^2^ and 3 × 3 cm^2^ field sizes, but more deviated for 4 × 4 cm^2^ and 6 × 6 cm^2^ field sizes for both the uncorrected and the corrected FOF especially for CC01. The clinical data yielded better agreement with calculation for field sizes of more than 1 × 1 cm^2^ field size. The set of small field data should match to the large field data. In this study, the % difference between measurement and calculation of clinical data was less than the three detectors where the commissioning data were 2 × 2 cm^2^ to 6 × 6 cm^2^ field sizes. The larger fields of more than 3 × 3 cm^2^ commissioning data using CC13 illustrated good result. The poor signal noise ratio of small size ion chamber makes it inferior to the large volume ion chamber for large field measurement. The unshielded diode, in contrast, tends to over response in large field measurement due to its energy dependency. The increasing contribution of lower energy scattering photon from larger field will soften the beam. For 1 × 1 cm^2^ field size which was calculated by AXB algorithm, the deviation occurred less than 1.5 % for PFD and EFD with both the uncorrected and the corrected FOF, but very high deviation in the uncorrected CC01. The FOF of each set of detector commissioning would influence the extrapolation calculation of FOF for the smaller fields less than 2 × 2 cm^2^ field size. The commissioning of FOF was acquired by field sizes defined by the jaws. The advanced techniques employed the MLC, the jaw tracking was used with 0.5 cm offset of the jaw from the MLC field edge in Varian TrueBeam machine. The verification of uncorrected FOF defined by the jaws and the MLC with 0.5 cm jaw offset measured by EDGE and CC13 are shown in Table 8. The MLC showed slightly more uncorrected FOF than the jaws, the differences were increased as the field sizes decreased up to 0.6%. Our MLC results agreed within 0.6% to Huq et al. who determined the uncorrected FOF of EDGE detector by MLC setting with jaw offset 0.5 cm from the field edge from 6×6 cm^2^ down to 2×2 cm^2^.^28^

**TABLE 8:** The comparison of uncorrected FOF between MLC and Jaws setting.

Side of square field (cm)	CC13		Ratio of MLC/Jaw	EDGE		Ratio of MLC/Jaw
	MLC	Jaw		MLC	Jaw	
10	1	1	1	1	1	1.000
6	0.925	0.922	1.003	0.921	0.918	1.003
4	0.870	0.865	1.006	0.865	0.863	1.003
3	0.837	0.833	1.005	0.833	0.830	1.004
2	0.795	0.790	1.006	0.799	0.794	1.006

FOF, field output factors; MLC, multileaf collimator.

The agreement of calculated MU among all commissioning datasets was characterized in terms of %SD. Overall, our research presented a significant reduction of %SD when the corrected FOF was incorporated to commissioning procedure. For symmetric field, %SD between both commissioning groups matched well from 6 × 6 cm^2^ to 2 × 2 cm^2^ field sizes. When the correction factors were applied to each detector, reduction of %SD was discovered until the smallest field size, which clearly demonstrates that the implementation of corrected FOF is able to reduce the discrepancy of calculated MU. For the field size smaller than 2 × 2 cm^2^ field size, the Eclipse™ TPS will calculate and extrapolate the dose according to the field output factors data set. If the corrected field output factors data are implemented, the calculated dose for smaller field sizes would be followed.

Similar trend was observed in IMRT‐SRS plans where the implementation of corrected FOF notably reduced %SD. IMRT technique employs variable intensity across multiple composite fields with the use of MLC to achieve a highly conformal dose distribution to the small and irregular tumor.^29^ Our results revealed that the reduction of %SD was more pronounced in case numbers 9 and 10 where multiple composite fields less than 2 × 2 cm^2^ field size were predominantly used. In case number 10, all commissioning datasets agreed well to compute MU in Eclipse™ within %SD less than 3%.

For VMAT‐SRS plans, it can be inferred that both commissioning groups exhibited a good agreement where the difference in average was approximately 0.3%. Even before corrected FOF were implemented for commissioning, the %SD of calculated MU was within 2.0%. This result draws a conclusion on either uncorrected or corrected FOF is used for small field commissioning, the number of calculated MU in VMAT does not vary much. This is related to the fact that VMAT technique delivers continuous beam to the target through the gantry rotation with less usage of multiple small segmented fields.^30^

The comparison of dose distribution between the calculation and measurement produced more deviation for IMRT plans with field sizes less than 3 × 3 cm^2^ due to the field output factors difference. The modulation factor seems to be less pronounced because of nonirregular tumor shape. The jaw tracking was implemented to both the IMRT and VMAT plans. The MLC for round shape tumors were not significantly modulated in the fluence map, so field output factors of the MLC can follow the field output factor defined by jaws. Therefore, no significant differences in MU were observed for all groups of datasets.

The case numbers 9 and 10 were selected to compare the dose distribution between calculated and measured of IMRT plans using MapCHECK2. The result of case number 9 (3 × 4 cm^2^) with gamma index of 2% dose difference and 2 mm distance to agreement (DTA) demonstrated that the calculated doses for corrected, uncorrected, and clinical data were comparable with 96.6% pass for all detectors. However, in case number 10 (2 × 2 cm^2^), the outcome showed datasets of corrected and uncorrected FOF by PFD and EFD passed 100%, which were different from CC01 for both corrected and uncorrected, as well as the clinical data set. They yielded the passing score of 88.9%. With the low resolution patient‐specific QA tool, no difference was observed for different data set of commissioning. The high‐resolution tools such as film should be studied for the next phase.

The limitation of this work was related to the commissioning procedure in Eclipse™ TPS. We were unable to integrate the field output factors less than 2 × 2 cm^2^. For smaller fields, it was left for Eclipse™ TPS to did extrapolation itself.

## 5 CONCLUSION

This study reported the number of monitor unit (MU) among commissioning datasets from three different detectors. The deviation reduced significantly when the correction factors based on IAEA/AAPM TRS 483 were implemented for small field commissioning 6 MV flattened photon beams in Eclipse™ treatment planning systems.

## CONFLICT OF INTEREST

No potential conflict of interest to disclose in this study.

## References

[1] International Commission on Radiation Units and Measurements (ICRU) Report No.91. Prescribing, Recording, and Reporting of Stereotactic Treatments with Small Photon Beams. Vol. 14 2017: Journal of the ICRU Oxford University Press p. 32–34.

[2] IAEA‐AAPM TRS 483 . Dosimetry of small static fields used in external beam radiotherapy: An International code of practice for reference and relative dose determination. 2017, Vienna, Austria: IAEA p. 9‐13. 10.1002/mp.13208 30247757

[3] Rustgi SN , Frye D . Dosimetric characterization of radiosurgical beams with a diamond detector. Med Phys. 1995;22:2117–2121. 8746721 10.1118/1.597655

[4] McKerracher C , Thwaites D . Assessment of new small‐field detectors against standard‐field detectors for practical stereotactic beam data acquisition. Phys Med Biol. 1999;44:2143–2160. 10495110 10.1088/0031-9155/44/9/303

[5] Das IJ , Downes MB , Kassaee A , Tochner Z . Choice of radiation detector in dosimetry of stereotactic radiosurgery‐radiotherapy. J Radiosurg. 2000;3:177–186.

[6] Cranmer‐Sargison G , Weston S , Sidhu NP , Thwaites DI . Experimental small field 6 MV output ratio analysis for various diode detector and accelerator combinations. Radiother Oncol. 2011;100:429–435. 21945858 10.1016/j.radonc.2011.09.002

[7] Wuerfel J . Dose measurements in small fields. Med Phys Int J. 2013;1:81–90.

[8] Benmakhlouf H , Sempau J , Andreo P . Output correction factors for nine small field detectors in 6 MV radiation therapy photon beams: a PENELOPE Monte Carlo study. Med Phys. 2014;41:041711. 24694131 10.1118/1.4868695

[9] Kairn T , Charles PH , Cranmer‐Sargison G , et al. Clinical use of diodes and micro‐chambers to obtain accurate small field output factor measurements. Australas Phys Eng Sci Med. 2015;38:357–367. 25744538 10.1007/s13246-015-0334-9

[10] Liu PZ , Suchowerska N , McKenzie DR . Can small field diode correction factors be applied universally? Radiother Oncol. 2014;112:442–446. 25441057 10.1016/j.radonc.2014.08.009

[11] Azangwe G , Grochowska P , Georg D , et al. Detector to detector corrections: a comprehensive experimental study of detector specific correction factors for beam output measurements for small radiotherapy beams. Med Phys. 2014;41:072103. 24989398 10.1118/1.4883795

[12] Alfonso R , Andreo P , Capote R , et al. A new formalism for reference dosimetry of small and nonstandard fields. Med Phys. 2008;35:5179–5186. 19070252 10.1118/1.3005481

[13] Derreumaux S , Boisserie G , Brunet G , Buchheit I , Sarrazin, T. Concerns in France over the Dose Delivered to the Patients in Stereotactic Radiation Therapy. In Standards, Applications and Quality Assurance in Medical Radiation Dosimetry (IDOS). Proceedings of an International Symposium. V. 1. 2011.

[14] Knöös T , Wieslander E , Cozzi L , et al. Comparison of dose calculation algorithms for treatment planning in external photon beam therapy for clinical situations. Phys Med Biol. 2006;51:5785. 17068365 10.1088/0031-9155/51/22/005

[15] Fogliata A , Lobefalo F , Reggiori G , et al. Evaluation of the dose calculation accuracy for small fields defined by jaw or MLC for AAA and Acuro sXB algorithms. Med Phys. 2016;43:5685–5694. 27782735 10.1118/1.4963219

[16] Fogliata A , Nicolini G , Clivio A , Vanetti E , Cozzi L . Dosimetric evaluation of Acuros XB Advanced Dose Calculation algorithm in heterogeneous media. Radiat Oncol. 2011;6:28. 21771317 10.1186/1748-717X-6-82 PMC3168411

[17] Tsuruta Y , Nakata M , Nakamura M , et al. Dosimetric comparison of Acuros XB, AAA, and XVMC in stereotactic body radiotherapy for lung cancer. Med Phys. 2014;41:081715. 25086525 10.1118/1.4890592

[18] Bush K , Gagne IM , Zavgorodni S , Ansbacher W , Beckham W . Dosimetric validation of Acuros® XB with Monte Carlo methods for photon dose calculations. Med Phys. 2011;38:2208–2221. 21626955 10.1118/1.3567146

[19] Han T , Mikell JK , Salehpour M , Mourtada F . Dosimetric comparison of Acuros XB deterministic radiation transport method with Monte Carlo and model‐based convolution methods in heterogeneous media. Med Phys. 2011;38:2651–2664. 21776802 10.1118/1.3582690 PMC3107831

[20] Kron T , Clivio A , Vanetti E , et al. Small field segments surrounded by large areas only shielded by a multileaf collimator: comparison of experiments and dose calculation. Med Phys. 2012;39:7480–7489. 23231297 10.1118/1.4762564

[21] García‐Garduño O , Rodríguez‐Ponce M , Gamboa‐deBuen I , et al. Effect of dosimeter type for commissioning small photon beams on calculated dose distribution in stereotactic radiosurgery. Med Phys. 2014;41:092101. 25186401 10.1118/1.4892176

[22] International Atomic Energy Agency, TECDOC 1583 . Commissioning of radiotherapy treatment planning systems : Testing for typical external beam treatment techniques 2008, Vienna, Austria: IAEA; 2008.

[23] Gibbons JP , Antolak JA , Followill DS , et al. Monitor unit calculations for external photon and electron beams: report of the AAPM Therapy Physics Committee Task Group No. 71. Med Phys. 2014;41:031501. 24593704 10.1118/1.4864244 PMC5148083

[24] IAEA TRS 398 . Absorbed dose determination in external beam radiotherapy: An international code of practice for dosimetry based on standards of absorbed dose to water. 2000, Vienna, Austria: IAEA 1‐183. p. 69.

[25] Das IJ , Cheng C‐W , Watts RJ , et al. Accelerator beam data commissioning equipment and procedures: report of the TG‐106 of the Therapy Physics Committee of the AAPM. Med Phys. 2008;35:4186–4215. 18841871 10.1118/1.2969070

[26] Das IJ , Ding GX , Ahnesjö A . Small fields: nonequilibrium radiation dosimetry. Med Phys. 2008;35:206–215. 18293576 10.1118/1.2815356

[27] Wang LL , Leszczynski K . Estimation of the focal spot size and shape for a medical linear accelerator by Monte Carlo simulation. Med Phys. 2007;34:485–488. 17388165 10.1118/1.2426407

[28] Huq MS , Hwang MS , Teo TP , Jang SY , Heron DE , Lalonde RJ . A dosimetric evaluation of the IAEA‐AAPM TRS483 code of practice for dosimetry of small static fields used in conventional linac beams and comparison with IAEA TRS‐398, AAPM TG51, and TG51 Addendum protocols. Med Phys. 2018;45:4257–4273. 10.1002/mp.13092 30009526

[29] Hall EJ . Intensity‐modulated radiation therapy, protons, and the risk of second cancers. Int J Radiat Oncol Biol Phys. 2006;65:1–7. 16618572 10.1016/j.ijrobp.2006.01.027

